# Meta-analysis of the efficacy of the pretreatment neutrophil-to-lymphocyte ratio as a predictor of prognosis in renal carcinoma patients receiving tyrosine kinase inhibitors

**DOI:** 10.18632/oncotarget.9836

**Published:** 2016-06-06

**Authors:** Ning Na, Jia Yao, Cailian Cheng, Zhengyu Huang, Liangqing Hong, Heng Li, Jiang Qiu

**Affiliations:** ^1^ Department of Kidney Transplantation, The Third Affiliated Hospital of Sun Yat-sen University, Guangzhou, Guangdong Province 510630, China; ^2^ Department of Hepatic Surgery, Liver Transplantation Center, The Third Affiliated Hospital of Sun Yat-sen University, Guangzhou, Guangdong Province 510630, China; ^3^ Department of Nephrology, The Third Affiliated Hospital of Sun Yat-sen University, Guangzhou, Guangdong Province 510630, China; ^4^ Department of Organ Transplant, The First Affiliated Hospital of Sun Yat-sen University, Guangzhou, Guangdong Province 510080, China

**Keywords:** renal cancer, neutrophil-to-lymphocyte ratio, prognosis marker, target therapy, meta-analysis

## Abstract

The data on the impact of the neutrophil-to-lymphocyte ratio (NLR) in metastatic renal cell carcinoma (mRCC) patients receiving tyrosine kinase inhibitors (TKIs) are inconsistent. We therefore performed a meta-analysis to assess the prognostic value of pretreatment NLR in patients treated with TKIs for mRCC. We searched the Embase, Medline, PubMed, Cochrane and ISI Web of Knowledge to identify clinical studies that had evaluated the association between the pretreatment NLR and prognosis in mRCC patients. Prognostic outcomes included overall survival (OS) and progression-free survival (PFS). Nine studies encompassing a total of 1091 participants were included. We found that a high NLR was an effective prognostic marker of both OS (pooled HR: 1.93, 95% CI: 1.35-2.77; P = 0.0003) and PFS (pooled HR: 2.12, 95% CI: 1.42-3.17; P = 0.0002). Subgroup analysis revealed that studies reporting a NLR ≥ 3 showed a more significant effect of NLR on both OS (pooled HR: 2.50, 95% CI: 1.99-3.14; P = 0.0003) and PFS (pooled HR: 2.17, 95% CI: 1.26-3.75). This meta-analysis suggests that high pretreatment NLR is associated with a poor prognosis in mRCC patients receiving TKI treatment.

## INTRODUCTION

Renal cell carcinoma (RCC) is the most common cancer of the kidney. Nearly half of RCC patients eventually develop metastatic disease (mRCC) [1, 2], and the 5-year survival rate among patients with mRCC remains poor. The molecular mechanisms underlying the pathogenesis of RCC has been widely investigated and has led to the development of several targeted agents [3]. In clinical trials, tyrosine kinase inhibitors (TKIs) such as sorafenib, sunitinib, bevasizumab and pazopanib have consistently prolonged progression-free survival (PFS) and, in some cases, overall survival (OS) among patients with metastatic RCC [4]. Because these agents have provoked marked changes in the management of RCC, new predictive and prognostic clinical markers are required.

The association between inflammation and cancer development has fostered an interest in the prognostic value of inflammatory factors [5, 6]. The neutrophil-to-lymphocyte ratio (NLR), an index defined as the absolute neutrophil count divided by the absolute lymphocyte count, has attracted the interest of investigators as a potential systemic inflammatory marker [7, 8]. Moreover, the NLR has been identified as an independent prognostic factor in several cancers. In RCC, for example, an increased preoperative or pre-treatment NLR is associated with a poor prognosis [9, 10], but the association between the NLR and treatment outcome in mRCC patients receiving VEGFR-TKIs has not been previously reviewed. Our aim, therefore, was to conduct a systematic review and meta-analysis to assess the predictive value of pre-treatment NLR in mRCC patients receiving VEGFR-TKIs.

## RESULTS

### Data retrieval

**Figure 1:**
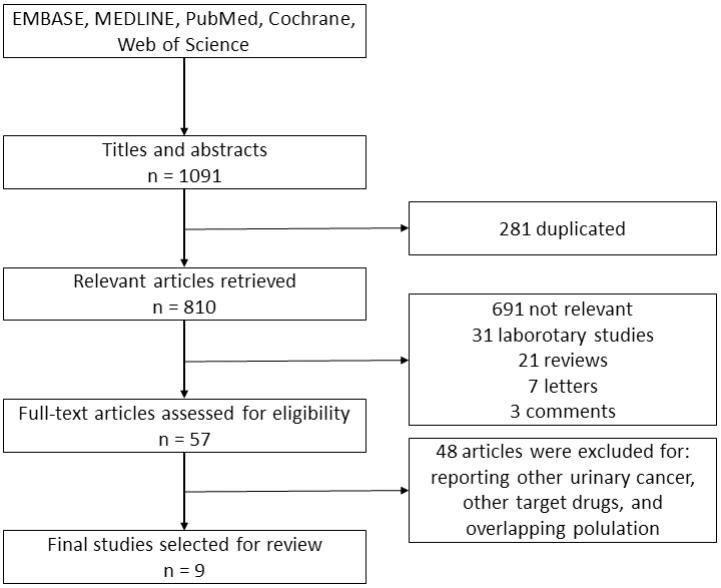
Literature screening flowchart

The work flow chart for this study is shown in Figure 1. The systematic search identified 1091 relevant references. Overall, 281 duplicated articles were removed. After screening titles and abstracts, we excluded 753 articles, including laboratory studies, meeting abstracts, reviews, letters and other articles irrelevant to our study. After assessing the full text, 48 additional articles were excluded. Ultimately, nine [11–19] retrospective cohort studies were included in the following meta-analysis.

### Study characteristics and quality assessment

The characteristics of the included studies and quality assessment results are shown in Table 1. The nine selected studies were published between 2013 and 2015. All trials were conducted in adult patients. Eight studies were conducted in Asian countries, including three in Turkey [11, 12, 14], two in China [17, 19], two in Israel [13, 15] and one in Korea [16]. The single remaining study was conducted in Italy [18]. Sample size for the included studies ranged from 23 to 373 patients, and a total of 1265 patients were included. The percentage of included males ranged from 63.4% to 80.7%, and the mean (median) age of the study patients ranged from 53 (median) to 64 (median) years. The NLR cutoff value ranged from 2.0 to 4.0.

**Table 1:** Characteristics of the included studies

Study	Year	Duration	Country	Sample size	Age (years)^*^	Male/Female	Tumor Histology		NLR cutoff value	Follow up (months)^*^	NOS
							Clear cell	Non-clear cell			
Cetin B, et al^11^	2013	2008.2–2011.12	Turkey	100	58 ± 10.6	NA	77	24	3.04	15 (1-53)	7
Dirican A, et al^12^	2013	2006.5-2011.3	Turkey	23	59 (43-76)	NA	18	5	3	13 (2-41)	6
Dana LS, et al^13^	2014	2006-2013	Israel	145	63.8 ± 11.2	92/53	113	32	3	49 ± 21	6
Gunduz S, et al^14^	2014	2009.5-2013.9	Turkey	45	63(IQR:41-90)	34/11	NA	NA	2	23.9^¶^	6
Keizman D, et al^15^	2014	2004.2-2013.3	Israel	278	62 ± 11.3	186/92	211	67	3	49 ± 21	6
Park YH, et al^16^	2014	2005.12-2011.12	Korea	109	61(IQR:49-67)	88/21	109	0	2.5	23.9 (IQR:10-35)	6
Wang HK, et al^17^	2014	2006.12-2011.3	China	41	53 (24-81)	33/8	34	7	4	NA	6
Santoni M, et al^18^	2015	2005.1-2014.6	Italy	151	64 (29-88)	99/52	151	0	3	51.6^⋆^	7
Zhang GM, et al^19^	2015	2006.12-2014.5	China	373	58 (17-90)	287/95	317	56	2.2	NA	6

* Values are given as mean±SD, median (range), or median (interquartile range).

¶ Value is given as mean.

⋆ Value is given as median.

NA=not available. NLR=Neutrophil to Lymphocyte Ratio. IQR=interquartile range. NOS=Newcastle-Ottawa Scale score.

### Study outcomes

In the included studies, a close relationship between NLR and cancer prognosis was detected. The majority of these studies were adjusted for potential confounders using the COX proportion hazard model; however, the HRs and 95% CIs were not explicitly stated in one study. Pooling the HR and 95% CIs extracted from the included studies revealed that a high NLR may be related to poor prognosis. After the data were combined, a high degree of heterogeneity was observed (p = 0.0001); thus, a random effects model was selected.

### Survival outcome

**Figure 2:**
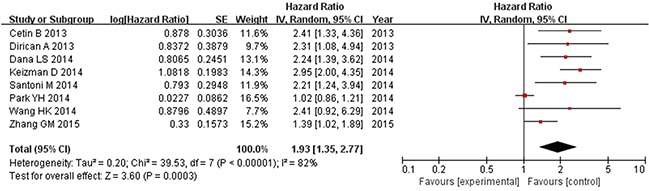
Overall survival based on the pretreatment NLR in mRCC patients receiving TKIs

**Figure 3:**
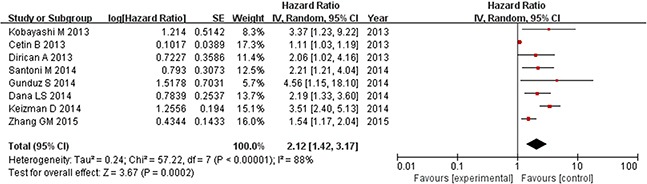
Progression-free survival based on the pretreatment NLR in mRCC patients receiving TKIs

Prognostic outcomes, including OS and PFS, were quantitatively synthesized. The results of the meta-analysis are displayed in Figures 2 and 3. Heterogeneity is illustrated in each forest plot. In Figure 2, OS values were available from eight [11–13, 15–19] studies on renal cell carcinoma. The synthesized hazard risk favored the low NLR patients (pooled HR: 1.93, 95% CI: 1.35-2.77; P = 0.0003; I^2^ = 82%), which meant that patients with a higher NLR had a greater mortality risk than those with a low NLR. Figure 3 shows that eight [11–15, 17–19] studies provided sufficient data on PFS outcome. The pooled results showed significant superiority of a low NLR in renal cancer (pooled HR: 2.12, 95% CI: 1.42–3.17; P = 0.0002; I^2^ = 88%). Because high I^2^ values were found in the pooled analysis, sensitivity analysis of the estimated outcomes was also performed. This analysis shows that no pooled outcomes (neither OS nor PFS) were altered by removing any included study, and the I^2^ value stayed above 80% except after removing the study of Park et al. [16] from the OS estimate (I^2^ decreased to 41%), or removing the study from Cetin et al. [11] from the PFS estimate (I^2^ decreased to 56%).

### Subgroup analysis

**Figure 4:**
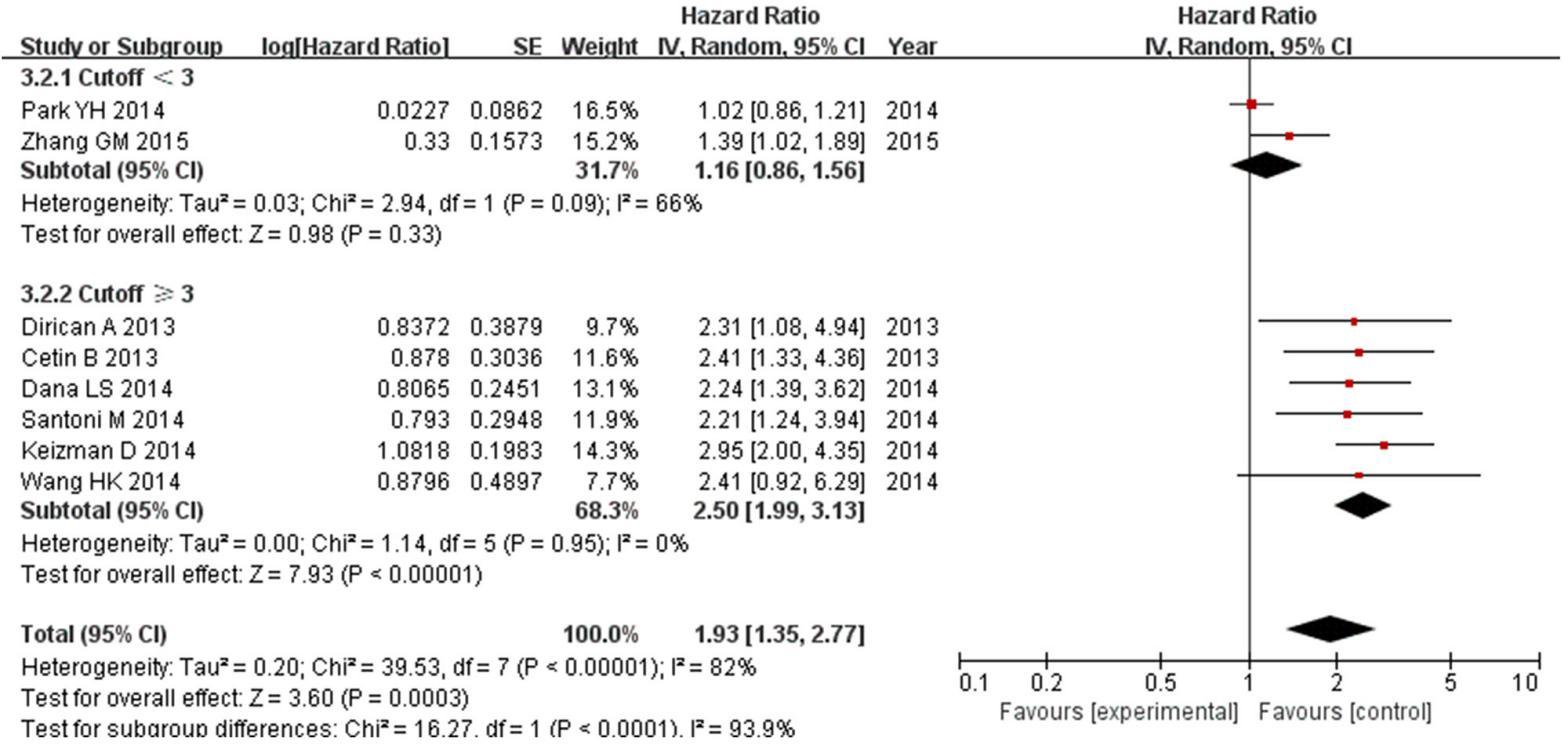
Subgroup analysis of pooled overall survival based on a NLR cutoff value

**Figure 5:**
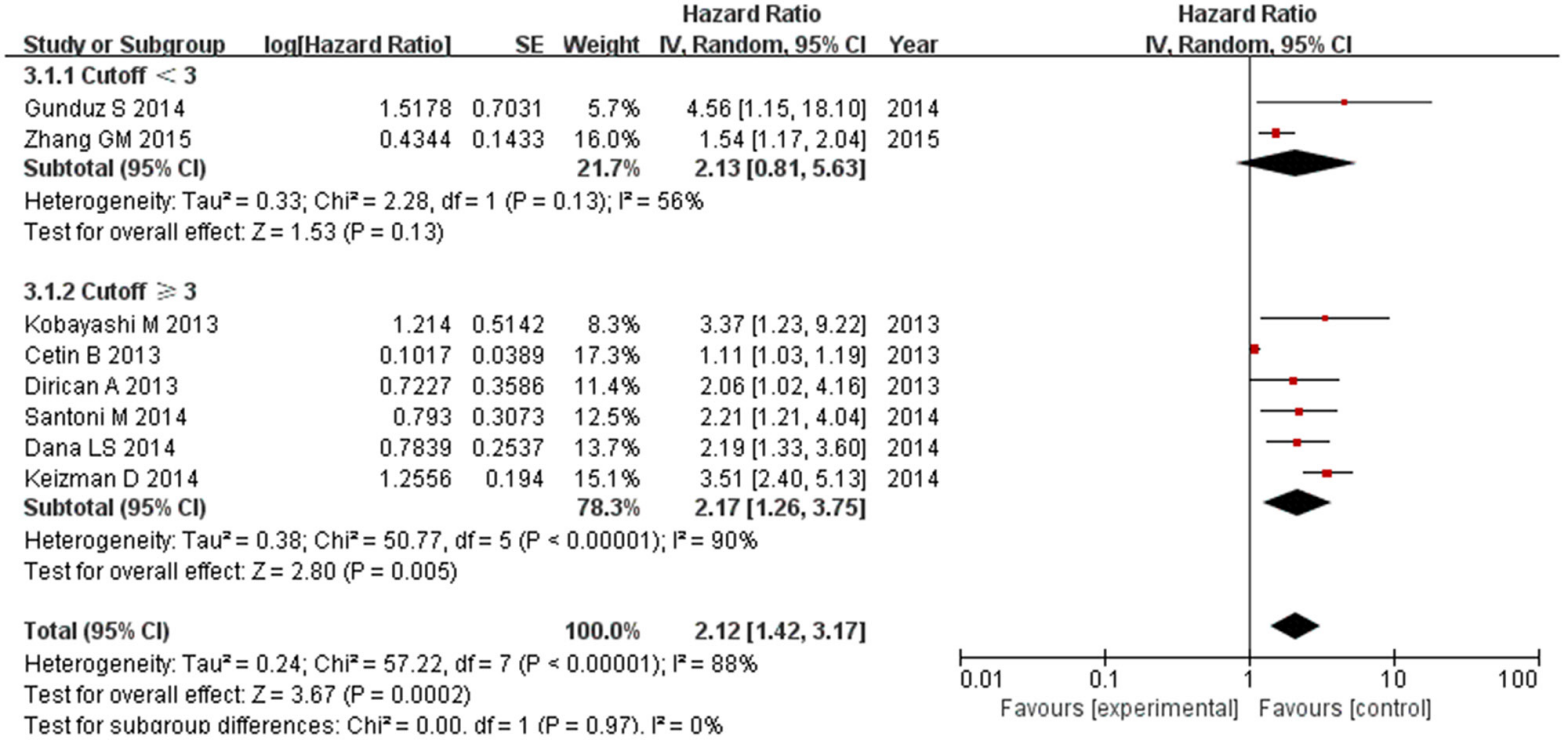
Subgroup analysis of pooled progression-free survival based on a NLR cutoff value

In a subgroup analysis, we evaluated the effect of NLR cutoff value on the results of pooled estimate. As shown in Figure 4, Pooled analysis of studies with lower NLR (< 3) revealed no significant difference in either OS (pooled HR: 1.16, 95% CI: 0.86-1.56; P = 0.33; I^2^ = 66%) or PFS (pooled HR: 2.13, 95% CI: 0.81-5.63; P = 0.13; I^2^ = 56%); between the two groups. By contrast, pooled analysis of those with higher NLR (≥ 3) showed a significant effect of NLR on both OS (pooled HR: 2.50, 95% CI: 1.99-3.13; P = 0.00001; I^2^ = 0%) and PFS (pooled HR: 2.17, 95% CI: 1.26-3.75; P = 0.005; I^2^ = 90%) (Figure 5).

### Publication bias

**Figure 6:**
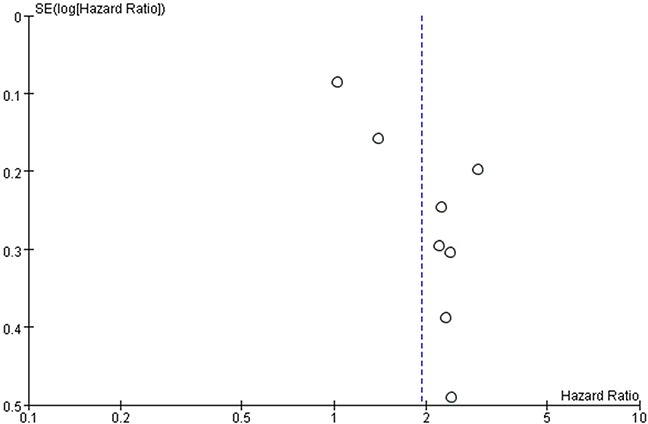
Funnel plots based on overall survival

**Figure 7:**
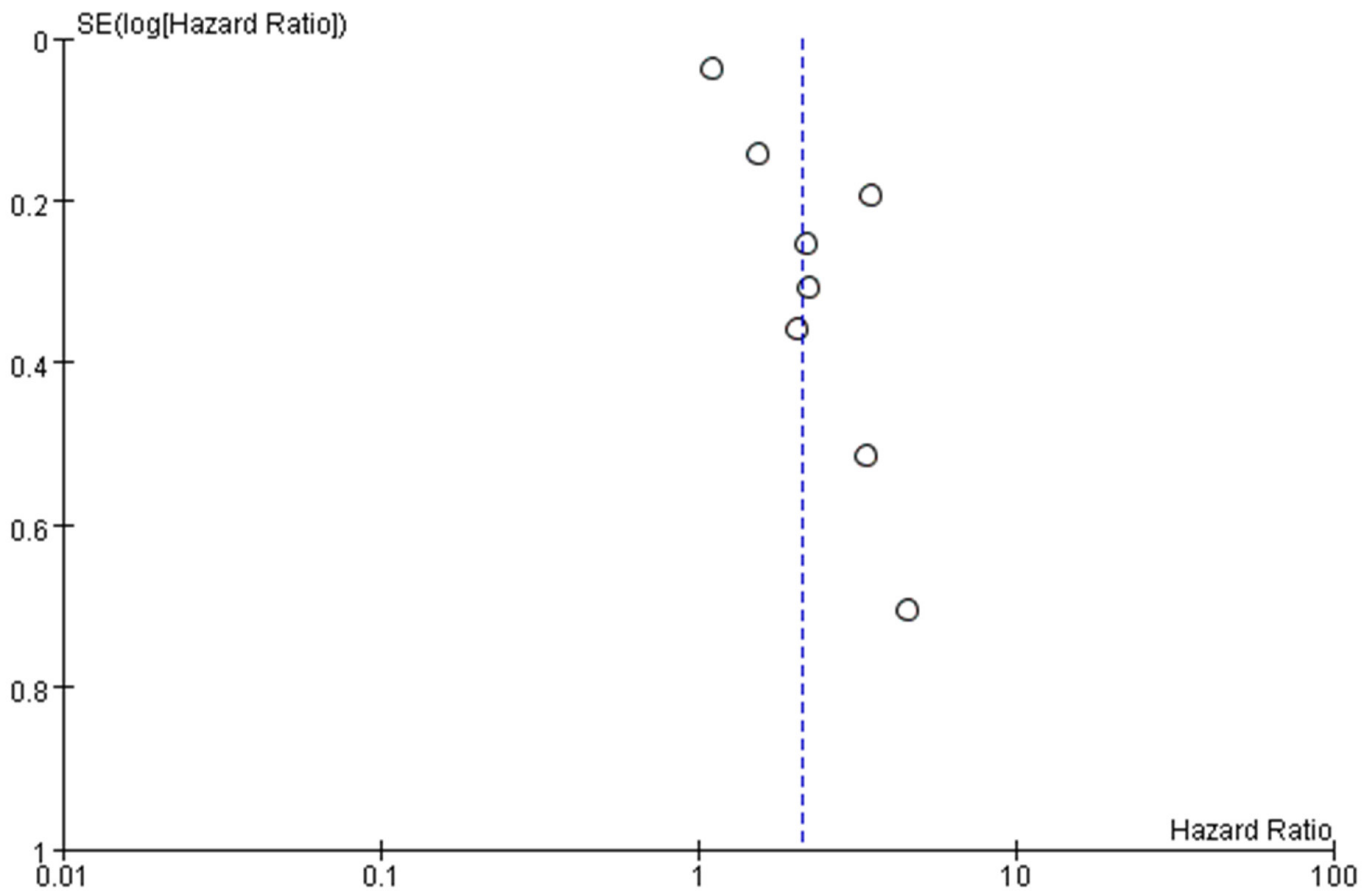
Funnel plots based on progression-free survival

Funnel plots of the studies used in the meta-analysis to evaluate OS and PFS are shown in Figures 6 and 7. The asymmetrically distributed plots indicated there was potential publication bias. However, because the number of included studies was just eight, the funnel plots may not be significant.

## DISCUSSION

Systemic treatment of mRCC has dramatically improved in recent years. TKIs are administered primarily as anti-angiogenic treatment of solid tumors [20]. Clinical trials showed that in advanced RCC, TKIs such as sorafenib, sunitinib and pazopanib exert consistent therapeutic effects that prolong both OS and PFS [21, 22]. However, these targeted agents require new prognostic markers. In this meta-analysis, we detected a favorable prognosis in mRCC patients who have a low NLR and use TKIs. Controlling for other clinical and demographic variables that might have affected survival, a high NLR was found to be an independent predictor of a poor prognosis in mRCC patients receiving TKIs therapy.

The interaction between inflammation and tumor development has been extensively studied for years [23], and tumor-promoting inflammation is now considered one of the enabling characteristics of cancer development [24]. In addition, it is now recognized that cancer progression is determined not only by the biologic characteristics of tumors, but also by the host's inflammation response [25], which is represented by the serum levels of white blood cells, neutrophils, lymphocytes and platelets, as well as by acute-phase proteins, such as C-reactive protein, and albumin [26]. Recently, various combinations of these factors, such the NLR, platelet-lymphocyte ratio (PLR), and Glasgow Prognostic Score (GPS), were reported to be useful prognostic indexes in certain solid tumors [26].

In cancer, neutrophils are a marker of inflammation related to the overproduction of cytokines in response to an increasing tumor burden or aggressive tumor biology. Emerging evidence also suggests that neutrophils are essential components of the tumor microenvironment [25, 27]. In RCC, increased blood neutrophil counts are reportedly associated with a poor prognosis both in localized cancers and metastatic cases [28, 29]. In contrast to neutrophils, lymphocytes are thought to confer an antitumor effect by inducing cell apoptosis, suppressing tumor growth and migration, and mediating cytotoxicity [30]. Furthermore, the NLR was shown to be a prognosis marker in both non-metastatic and metastatic RCC patients [9]. Because this parameter is simple and easy to measure using widely available standardized assays, it has the potential to serve as a prognosis marker that cheap, widely available, and easily standardized for clinical management of renal cancer.

The data on the implications of the NLR in mRCC patients receiving TKIs are inconsistent. We therefore conducted a meta-analysis to explore the prognostic value of the NLR in these patients. Our analysis revealed that the NLR is predictive of prognosis in mRCC patients using TKIs. Given that TKIs such as sorafenib, sunitinib, and pazopanib exert both anti-angiogenic and immunomodulatory effects, including effects on neutrophil migration and T lymphocyte-DC cross-talk [31–33], the implications of the NLR in mRCC patients receiving targeted therapy may have more significance than in the RCC patient population as a whole.

The quality of the included studies was close, as all the studies showed inadequacy in the area of selecting the NLR ratio and the comparability of the groups. All of the included studies used a dichotomous NLR ratio to evaluate its prognostic value. The cutoff values were determined by the investigators and differed among the studies. It appears the NLR cutoff value was calculated in each study to acquire the most significant effect, from which the final significance of the outcomes seemed to be created. Additionally, because none of the studies used a matched design, the comparability of the clinicopathological factors between patients in different groups was poor. Because of these deficiencies, although we are able to conclude that the pooled estimate of available studies indicates that a higher NLR is associated with a poor prognosis, whether the NLR can serve prospectively as a clinical marker of prognosis will need further investigation.

There are other limitations to this study. First, all the included studies in our meta-analysis were retrospective. In observational studies, selection bias is impossible to avoid. Second, although our subgroup analysis revealed there was no heterogeneity in the pooled HR for OS, there was high heterogeneity in the pooled HR for PFS. Third, the funnel plot showed high standard errors between studies with large sample sizes and those with small sample sizes, which suggests selective outcome reporting or publication bias. However, because the number of studies was only eight, the significance of the funnel plot may not be significant.

In summary, our analysis of currently available clinical evidence indicates that a high pretreatment NLR is associated with a poor prognosis in mRCC patients receiving TKIs treatment. Based on the limitations of both the studies evaluated and our meta-analysis, further well-designed studies are need to draw a more definite conclusion as to the clinical significance of NLR as a prognosis marker. In future studies, we recommend using a prospective and matched study design, and using a continuous NLR variable rather than a categorical variable.

## MATERIALS AND METHODS

### Study identification and selection

A literature search was performed in October 2015 without restriction on region or publication type. Five electronic databases (PubMed, MEDLINE, EMBASE, Web of Science, and the Cochrane Library) were searched to identify possible articles relevant to the topic of interest. The Mesh terms and text words “neutrophil,” “lymphocyte,” “renal cancer” and “urinary cancer” were used to find eligible studies. Two reviewers (Na N. and Yao J.) independently screened the search results for titles and abstracts. The references cited in all full-text articles were also assessed for additional potential relevant articles. If either reviewer felt a title and abstract met the following eligibility criteria – 1) prospective or retrospective studies on the role of NLR in predicting prognosis in mRCC patients; 2) patients received TKI therapy for mRCC; 3) pretreatment NLR was measured before administering TKIs; 4) the hazard ratio (HR) for progression-free survival (PFS) or overall survival (OS), along with their 95% confidence intervals (CIs) or p values were available – the full text of the study was further screened for eligibility. In articles reporting cancer-specific survival, the cancer-specific survival was considered to approximate OS. Peer-reviewed publications that met the criteria were eligible for inclusion. When multiple reports describing the same population were published, the most recent or complete report was used. Studies were excluded from the analysis included duplicated studies; duplicated reported data; laboratory studies; experimental animal studies; letters; review articles; case reports; and abstracts only.

### Quality assessments

The quality of each trial was evaluated using the “Newcastle-Ottawa Scale (NOS)” for cohort studies, which considered three factors (http://www.ohri.ca/programs/clinical_epidemiology/oxford.asp.), patient selection, comparability of the study groups and assessment of outcome, with a total score of 0 to 9; studies achieving a score of 6 or higher were considered to be of high quality. The quality of each eligible study was evaluated independently by two reviewers (Na N. and Yao J.) based on the methodology used. Corresponding authors of eligible studies were contacted to clarify any questions about the methodology so as to assess each study as accurately as possible. If there was any disagreement, a third author would arbitrate.

### Data abstraction

Two investigators (Na N. and Chen C.) reviewed the full manuscripts independently using standardized data-abstraction forms, which included the following information: author, year of publication, state of the research, sample size, age and gender of patients, tumor type (clear cell or non-clear cell), metastasis site, cut-off value of NLR, follow-up time, prognostic outcomes and statistic model. The HR was preferred for measuring the prognostic outcome, since it is time-to-event data. In studies showing only survival or mortality curves, the HRs and 95% CIs were estimated using the methods described by Tierney et al. [34], or the corresponding author was contacted to obtain the original data or results.

### Statistical analysis

Review Manager Version 5.3 software (Version 5.3 for Windows, The Cochrane Collaboration, 2014) was used to carry out the pooled analysis. HR was selected as the effect to measure prognosis outcomes, which was reported along with the corresponding 95%CI. Values of P < 0.05 were considered statistically significant. Cochran's Q test and Higgins I^2^ statistic were used to assess heterogeneity across studies. Studies with a P < 0.1 and/or I^2^ > 50% were considered indicative of large heterogeneity. The random-effects model was used if there was heterogeneity between the studies; otherwise, the fixed-effects model was used. Additionally, Egger's test and Begg's test were used to evaluate publication bias (STATA v. 11.0, StataCorp, College Station, TX, USA).
